# Vitamin D and Bone: A Story of Endocrine and Auto/Paracrine Action in Osteoblasts

**DOI:** 10.3390/nu15030480

**Published:** 2023-01-17

**Authors:** Marjolein van Driel, Johannes P. T. M. van Leeuwen

**Affiliations:** Department of Internal Medicine, Room Ee585c, Erasmus MC, Dr. Molewaterplein 40, 3015 GD Rotterdam, The Netherlands

**Keywords:** vitamin D metabolism, vitamin D receptor, bone, osteoblasts, differentiation and mineralization

## Abstract

Despite its rigid structure, the bone is a dynamic organ, and is highly regulated by endocrine factors. One of the major bone regulatory hormones is vitamin D. Its renal metabolite 1α,25-OH2D3 has both direct and indirect effects on the maintenance of bone structure in health and disease. In this review, we describe the underlying processes that are directed by bone-forming cells, the osteoblasts. During the bone formation process, osteoblasts undergo different stages which play a central role in the signaling pathways that are activated via the vitamin D receptor. Vitamin D is involved in directing the osteoblasts towards proliferation or apoptosis, regulates their differentiation to bone matrix producing cells, and controls the subsequent mineralization of the bone matrix. The stage of differentiation/mineralization in osteoblasts is important for the vitamin D effect on gene transcription and the cellular response, and many genes are uniquely regulated either before or during mineralization. Moreover, osteoblasts contain the complete machinery to metabolize active 1α,25-OH2D3 to ensure a direct local effect. The enzyme 1α-hydroxylase (*CYP27B1*) that synthesizes the active 1α,25-OH2D3 metabolite is functional in osteoblasts, as well as the enzyme 24-hydroxylase (*CYP24A1*) that degrades 1α,25-OH2D3. This shows that in the past 100 years of vitamin D research, 1α,25-OH2D3 has evolved from an endocrine regulator into an autocrine/paracrine regulator of osteoblasts and bone formation.

## 1. Introduction

The skeleton plays a fundamental role in the human body by providing structural support and allowing movement. Moreover, it has a protective role for vital internal organs and stem cells, is a source for mineral and growth factors, and is the center of regulatory pathways. Bone is highly dynamic and undergoes continuous remodeling throughout life; it can repair itself. To illustrate this, damaged or (micro)fractured areas are removed by osteoclastic bone resorption, which is followed by new bone formation by osteoblasts (bone remodeling). Bone formation is characterized by secretion of an extracellular proteinaceous matrix, which is subsequently mineralized. Bone remodeling is tightly controlled by an interplay of local, bone and bone marrow-derived factors (e.g., cytokines, growth factors, chemokines) and endocrine factors. One of these endocrine factors is the seco-steroid 1α,25-dihydroxyvitamin D_3_ (1α,25-OH_2_D_3_). 1α,25-OH_2_D_3_ can affect bone in a direct as well as an indirect manner [1,2,3]. The indirect effect occurs via control of calcium reabsorption in the kidney and absorption in the intestine, as well as via control of parathyroid hormone production. Although rickets and osteomalacia were prevented in vitamin D receptor (*VDR*) knockout mice fed with a rescue diet that contained high levels of calcium and phosphorus, not all bone changes were rescued, indicating the importance of a direct role for 1α,25-OH_2_D_3_ in bone metabolism [4,5,6]. The presence of VDRs in cells of the osteoblast lineage [7,8] enables direct effects of 1α,25-OH_2_D_3_ on bone metabolism. *VDR* expression in osteoblasts can be regulated by 1α,25-OH_2_D_3_ itself, as well as by other factors including parathyroid hormone, glucocorticoids, transforming growth factor-β, and epidermal growth factor [9,10,11,12,13]. Transgenic mice specifically overexpressing the *VDR* in osteoblasts have increased trabecular bone volume and increased bone strength, supporting an anabolic effect of 1α,25-OH_2_D_3_ [14]. This observation was confirmed in a study using mice with a different genetic background [15]. Interestingly, a study with global *VDR* knockout mice [5] knockout mice reported a similar phenotype, with increased trabecular thickness and increased osteoid volume and osteoblast numbers, suggesting an inhibitory effect of 1α,25-OH_2_D_3_ on bone formation. This was supported by data from an osteoblast-specific *VDR* knockout mouse study [16]. In this latter study, the bone effect appeared to be via reduced bone resorption. The effects on bone may be related to overall levels of calcium intake [17], but whether this explains the apparent opposite effects in murine studies remains to be established. Nevertheless, these observations support a direct effect of 1α,25-OH_2_D_3_ on bone metabolism via osteoblasts. There is less consensus on VDR expression in osteoclasts. Genomic deletion of the *VDR* in osteoclasts did not impact the positive effect of a 1α,25-OH_2_D_3_ analog (eldecalcitol) on bone mass [7]. This is supported by Verlinden et al., who reported that *VDR*s in osteoclast precursors are not essential to maintain bone homeostasis [18]. It was concluded that 1α,25-OH_2_D_3_ regulates osteoclasts indirectly via cells of the osteoblast lineage. In the current review, we will focus on 1α,25-OH_2_D_3_ in osteoblast function and bone metabolism.

## 2. Literature Search Strategy

We built on our pre-existing literature database and expanded this with a new search from 2016 until October 2022. With the support of the Erasmus MC Medical Library Literature Search Service, the search strategy was developed and executed. Supplemental Figure S1 shows in detail the search strings used. In this way, we obtained a list of 2713 publications on vitamin D. From this dataset, we excluded 2583 clinical and (genetic) epidemiological association studies and focused on 128 bone-related molecular and cellular studies. Two publications appeared to be retracted after the search was performed.

## 3. Osteoblasts

Osteoblasts originate from mesenchymal stromal cells via a tightly controlled differentiation process. The eventual fate of osteoblasts is three-fold, either to become lining cells that cover the bone surface, or to become embedded in the extracellular matrix as osteocytes, or to die via apoptosis. 

### 3.1. Proliferation and Apoptosis

The data on 1α,25-OH_2_D_3_ effects on osteoblast proliferation are variable. Inhibition [19,20,21,22,23,24,25,26,27], as well as stimulation [20,28] or no effect [29,30] on the proliferation of osteoblasts of mouse, rat, and human origins have been reported. Effects on cell viability [31] and apoptosis [32,33] have also been documented. Although different directions in effect have been observed, these data demonstrate direct effects of 1α,25-OH_2_D_3_ on osteoblast proliferation and survival. The direction of effect may depend on the timing of treatment, dosage, origin, and environment of the osteoblasts [27,34,35,36]. 

### 3.2. Differentiation

**Figure 1:**
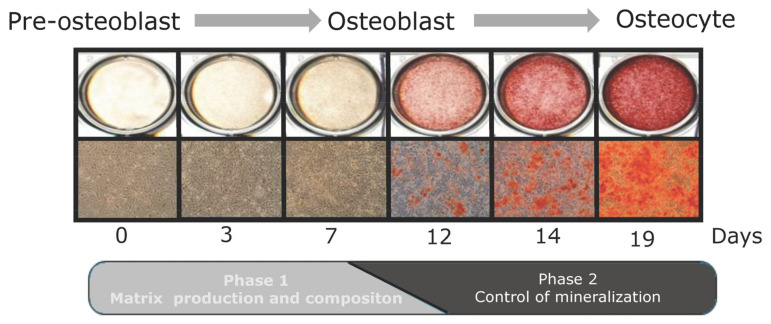
Alizarin red staining of osteoblast culture exemplifying the pre-mineralization and mineralization phases. Red staining shows mineralization. Details on cell culture and Alizarin red staining procedures can be found in Woeckel et al. [99]. Adapted with permission from Eijken, M., Koedam, M., van Driel, M., Buurman, C.J., Pols, H.A.P., van Leeuwen J.P.T.M. The essential role of glucocorticoids for proper human osteoblast differentiation and matrix mineralization. Mol Cell Endocrinol 2006, 248(1–2):87–93. https://doi.org/10.1016/j.mce.2005.11.034. 2006, J.P.T.M. van Leeuwen.

Immature mesenchymal stromal cells differentiate into osteoblasts that produce extracellular matrix proteins, enzymes, and matrix vesicles involved in the mineralization of the extracellular matrix produced (Figure 1). It has been demonstrated that 1α,25-OH_2_D_3_ impacts all of these processes [3,37,38]. 1α,25-OH_2_D_3_ stimulation of differentiation has been shown in all in vitro studies using human osteoblasts, human mesenchymal stem cells, and osteogenic-induced pluripotent stem cells [30,39,40,41,42,43,44,45,46]. Most studies with rat osteoblasts resemble these studies using human osteoblasts and show increased differentiation [29,47,48]. Studies with mouse osteoblasts are more diverse. These studies show inhibition [49,50], as well as stimulation of osteoblast differentiation by 1α,25-OH_2_D_3_ [51]. The definitive explanation for the discrepancies in 1α,25-OH_2_D_3_ effects between, on the one hand, mouse osteoblast cultures, and on the other hand, between mouse and human/rat osteoblast cultures, is absent; however, several explanations can be put forward. The source of osteoblasts may play a role. Different sites of the skeleton differ in origin and bone formation, such as enchondral (long bones) and intramembranous (calvaria) sites. 1α,25-OH_2_D_3_ did not affect osteoblasts from cortical bone, and inhibited differentiation of calvaria-derived cells [52,53]. Furthermore, within one skeletal element, differences in osteoblast regulation are observed. A recent study reported differences between periosteal- and bone-marrow-derived osteoblasts in cortical bone [54]. Whether this fully explains the diverse effects observed is not clear, but it shows the importance of origin for the eventual activity and regulation. This may also relate to stage of osteoblast differentiation, donor age, culture conditions, etc., which have been shown to relate to 1α,25-OH_2_D_3_ action [17,47,55,56]. Furthermore, differences may be species-intrinsic, and may have a genomic explanation. 1α,25-OH_2_D_3_ increases *RUNX2* and *BGLAP* (osteocalcin) gene expressions in human osteoblasts, while in murine osteoblasts, 1α,25-OH_2_D_3_ treatment inhibits the gene expressions of *RUNX2* and *BGLAP* [43,57,58,59,60,61].

A picture that emerges from all in vitro osteoblast data is that the osteoblast (micro)environment is a determinant of the eventual outcome of 1α,25-OH_2_D_3_ action. The extracellular milieu (growth factors, cytokines, matrix proteins, ions (calcium/phosphate), and other signaling molecules) and the intracellular milieu (e.g., the insulin-like growth factor binding protein-6) are important for the eventual effect of 1α,25-OH_2_D_3_ [62,63]. For example, interactions with transforming growth factor-β, insulin-like growth factor, bone morphogenetic proteins, and interferon have been demonstrated [64,65,66,67,68,69]. Consequently, the absence or presence of these, but potentially other factors as well, can modulate 1α,25-OH_2_D_3_ action and determine the eventual response. An example of interaction with other intracellular regulatory pathways is Wnt signaling. Wnt signaling plays an important role in osteoblast differentiation and bone formation. An interplay between 1α,25-OH_2_D_3_ and Wnt signaling has been described [70,71,72,73,74]. 

Osteoblast differentiation, bone matrix production, and mineralization, as part of bone formation, are high energy-demanding processes [75,76,77]. Regulation of energy metabolism impacts osteoblast differentiation and bone formation [78,79,80]. Vitamin D and energy metabolism have been discussed in relation to obesity and metabolic syndrome [81] and to cancer [82,83,84], but data on vitamin D and energy metabolism in the context of osteoblast differentiation remain limited. Forkhead Box O (*FoxO*) transcription factors are regulated by 1α,25-OH_2_D_3_ in murine MC3T3 osteoblasts. *FoxO3a* is upregulated, *FoxO1* is downregulated, and *FoxO4* is unchanged after 1α,25-OH_2_D_3_ treatment. si-RNA knockdown of the *FoxO*s did not change 1α,25-OH_2_D_3_ inhibition of proliferation [85]. Unfortunately, the effect on differentiation was not reported. Changes in *FoxO* expression were coupled to increase in reactive oxygen species accumulation, which may be linked to cellular metabolism and bone formation [75,80,86]. Glucose, insulin, and 1α,25-OH_2_D_3_ regulation of osteoblast proliferation, alkaline phosphatase activity, and production of (uncarboxylated) osteocalcin have been studied in isolated rat osteoblasts, but unfortunately, no coupling to mineralization was made [87]. Nevertheless, these data, together with those on interactions between vitamin D and *PPARγ* signaling in osteoblast differentiation [88], support that control of energy metabolism can be a vitamin D target in bone formation and mineralization. 

### 3.3. Mineralization

Mineralization can be divided into two phases. In the first phase, formation of hydroxyapatite (HA) crystals takes place in nano-sized extracellular matrix vesicles produced by osteoblasts. In the second phase, HA is propagated outside these vesicles, with a resulting buildup of mineral in the extracellular matrix [89,90]. Calcium and phosphate concentrations increase inside matrix vesicles via involvement of specific proteins, and when the solubility product of calcium and phosphate is exceeded, mineral deposits are formed inside the extracellular vesicles and the second phase of mineralization starts with the release of the preformed HA crystals [90,91]. Proteomic analyses of extracellular matrix vesicles revealed many proteins with a potential role in mineralization [92,93]. Gene profiling studies also identified novel regulators of osteoblast matrix mineralization [94]. 

Mineralization is controlled by a balanced action of promoters and inhibitors. Alkaline phosphatase and bone sialoprotein are important promoters [95,96]. Alkaline phosphatase increases the phosphate concentration in matrix vesicles by hydrolyzing inorganic pyrophosphate. Pyrophosphate is an inhibitor of mineralization; consequently, alkaline phosphatase also decreases the level of this inhibitor. Pyrophosphatase phosphodiesterase 1 (NPP1, encoded by the gene *ENPP1*) and ankylosis protein (ANK) are involved in inhibiting mineralization. NPP1 generates pyrophosphate, and the transmembrane protein ANK allows pyrophosphate to pass through the plasma membrane to the extracellular matrix; thus, HA formation is inhibited in the extracellular vesicles [97,98]. 1α,25-OH_2_D_3_ stimulates mineralization via direct action on osteoblasts [68,88,99]. 1α,25-OH_2_D_3_ can influence the mineralization process via gene expression and matrix vesicle production. Gene expression profiling studies demonstrated that the 1α,25-OH_2_D_3_ effect is not likely primarily due to changes in the expression of extracellular matrix genes, and thereby to changes in composition of the extracellular matrix [99]. Studies on the expression and production of procollagen type I by human osteoblasts showed stimulation [100,101] as well as no effect [101,102,103,104], or inhibition [105].

It is postulated that vitamin D may enhance mineralization by stimulating both NPP1, generating pyrophosphate, and alkaline phosphatase, generating phosphate from pyrophosphate [106]. This involves acceleration of the production of alkaline phosphatase-positive matrix vesicles, leading to enhanced formation and deposition of HA crystals, and eventually mineralization. This direct effect of vitamin D occurred in the period prior to the onset of mineralization, and also involved accelerated extracellular matrix maturation [99]. Interestingly, treatment with vitamin D after initiation of mineralization did not affect mineralization. This supports the above-described osteoblast differentiation stage dependency of the 1α,25-OH_2_D_3_ effect. A study by Yajima et al. described the significance of 1α,25-OH_2_D_3_ for osteocytic perilacunar mineralization [107].

1α,25-OH_2_D_3_ also directly stimulates the production of inhibitors of mineralization. VDR-dependent 1α,25-OH_2_D_3_ expression of *ENPP1* and *ANK* in murine osteoblasts led to an increase in the mineralization inhibitor pyrophosphate [108]. 1α,25-OH_2_D_3_ also stimulates activin A expression in human osteoblasts. Treatment with the activin A blocker follistatin enhanced vitamin-D-induced mineralization of human osteoblasts [109]. 1α,25-OH_2_D_3_ also increases the expression of osteopontin, which is shown to inhibit mineralization. These observations may provide a fine-tuning mechanism to prevent excessive mineralization of bone. 1α,25-OH_2_D_3_ induction of carboxylated osteocalcin may be in line with this. 1α,25-OH_2_D_3_-stimulated mineralization is enhanced by blocking osteocalcin carboxylation by warfarin [109]. The interaction of 1α,25-OH_2_D_3_ with other factors, as described above, also holds for mineralization, for example, the interaction with DKK1, the inhibitor of Wnt signaling [74].

The counterbalance of bone formation and mineralization by osteoblasts is bone resorption by osteoclasts. In the healthy skeleton, these processes are in balance, securing healthy and strong bones. The osteoblasts/osteocytes are the major regulators of osteoclast formation and action via production of the stimulating factor RANKL, and the RANKL inhibitor, osteoprotegerin (OPG). 1α,25-OH_2_D_3_ influences the RANKL/OPG ratio, and thereby also impacts bone resorption [110,111,112,113]. 1α,25-OH_2_D_3_ is involved at both the bone formation and the bone resorption sides of the balance, and is an important player in maintaining healthy bones via direct effects on bone, in addition to indirect effects via calcium and phosphate homeostasis [114].

### 3.4. Gene Expression

The basis of all cellular effects of 1α,25-OH_2_D_3_ involves VDR-mediated transcriptional regulation. The VDR is a member of the nuclear receptor family. Upon binding to 1α,25-OH_2_D_3_, the VDR heterodimerizes with the retinoic X receptor (RXR), and binds as a dimer to the vitamin D response element (VDRE) in the DNA to regulate gene expression [115]. Over the years, many studies have unraveled the molecular fundamentals of 1α,25-OH_2_D_3_ transcriptional regulation. Examples and information can be found in these publications and references therein [116,117,118]. In a previous publication, we discussed 1α,25-OH_2_D_3_ and gene transcription in osteoblasts [38]. This will not be repeated or discussed in detail in this review. 

A factor that may determine the transcriptional effect of 1α,25-OH_2_D_3_ effect is not only the basal level of gene expression [51,119], but also the stage of osteoblast differentiation [99]. Studies with rat osteoblasts in the early 1990s showed already that effects of 1α,25-OH_2_D_3_ on osteoblasts may depend on the osteoblast differentiation phase [119]. An example is the 1α,25-OH_2_D_3_ stimulation of phosphaturic hormone fibroblast growth factor 23 (FGF23) only in late-stage differentiation osteoblasts and osteocytes [120,121]. FGF23 is a hormone that acts in the kidney to enhance phosphate excretion, and suppresses 1α,25-OH_2_D_3_ synthesis by inhibiting 1α-hydroxylase (CYP27B1), forming an important loop in the regulation of mineralization [122,123]. Vitamin D signaling in osteocytes [124] is further supported by the 1α,25-OH_2_D_3_ regulation of *PHEX* (phosphate-regulating neutral endopeptidase, X linked), which suppresses FGF23 transcription [125]. 

The various osteoblast differentiation stages actually reflect different functional stages of the osteoblast, e.g., proliferation, extracellular matrix production, mineralizing and mechanosensing. It is important to keep in mind the osteoblast differentiation stage when studying 1α,25-OH_2_D_3_ effects, as this may be an important determinant of the eventual effect (e.g., stimulation or inhibition) on gene transcription and subsequent cellular responses and bone formation. The relationship between the osteoblast differentiation stage and 1α,25-OH_2_D_3_ gene expression control was shown by Woeckel et al. [99]. 1α,25-OH_2_D_3_ changed the expression of different sets of genes in the phase before the onset of mineralization, and during the mineralization. For this review, we performed a reanalysis of this gene profiling study [99] with the 2022 updated annotation. Comparison of transcripts regulated (i.e., two-fold up or down) in the phase before and after the start of mineralization (Figure 1) demonstrated that only 2.5% (18 out of the 721 regulated transcripts) were regulated in both phases (Table 1). The gene symbols of the transcripts regulated in both phases are shown in Table 2. To focus in more detail on phase-specific gene expression, we next selected the transcripts that were uniquely regulated in either the pre-mineralization or in the mineralization phase [99]. In this regard, the transcripts should be at least two-fold up- or downregulated in one phase (either pre-mineralization or mineralization phase), and not regulated (fold change on average between 0.8 and 1.2) in the other phase (either the mineralization or pre-mineralization phase). Table 3 shows the number of transcripts uniquely regulated in either of these phases, and Table 4 reports the gene symbols belonging to these transcripts. This binary comparison of pre-mineralization and mineralization phases is not absolute and does not mean that further zooming in on specific phases of osteoblast differentiation will not reveal other sets of vitamin-D-regulated genes. However, it does support the notion that vitamin D gene regulation during osteoblast differentiation and mineralization displays temporal dynamics, and it does show that for proper interpretation of vitamin D effects, knowledge on the differentiation and functional stage of cells and tissues is important. This knowledge can explain the apparent differences in 1α,25-OH_2_D_3_ effects that have been reported. 

## 4. Vitamin D Metabolism

**Figure 2:**
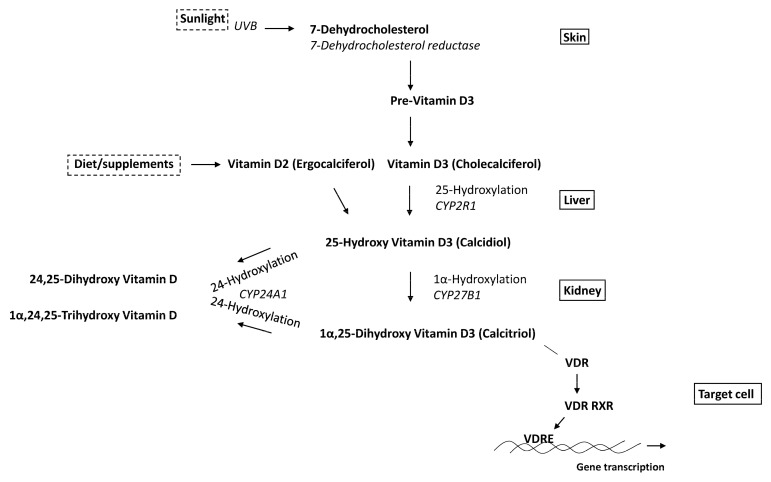
Schematic representation of classic vitamin D metabolism and signaling pathway. Either from sunlight or food, vitamin D is converted via enzymatic reactions in the liver and kidney into its active metabolite, 1α,25-OH_2_D_3_, which binds to the VDR. Gene activation follows after binding of the vitamin D/receptor complex to vitamin D response elements (VDREs) in target genes.

Metabolism, synthesis of the active form of 1α,25-OH_2_D_3_ as well as its inactivation, has been an important research topic since the identification of vitamin D. This has contributed to the understanding of the initiation and termination actions of vitamin D and its endocrine function. Figure 2 shows the classical vitamin D metabolism pathway. Serum levels of 1α,25-OH_2_D_3_ are determined by the activity of the renal enzyme 1α-hydroxylase (CYP27B1). 24-Hydroxylase (CYP24A1) is the first step of a 1α,25-OH_2_D_3_ inactivation cascade present in all target tissues. In the next sections, we discuss CYP27B1 and CYP24A1 in osteoblasts. 

### 4.1. CYP27B1

In the late 1970s and early 1980s, reports were already coming out that in tissues other than the kidney, 1α,25-OH_2_D_3_ can be synthesized. Cells isolated from chicken calvaria [126] and human osteosarcoma cells, as well as bone cells isolated from an ileac crest biopsy [127], can produce 1α,25-OH_2_D_3._. Its functional significance in human osteoblasts was shown by the fact that inhibition of 1α-hydroxylase activity by ketoconazole blocked the 25(OH)D_3_ induction of *CYP24A1* and osteocalcin expression [30]. This was supported by studies on siRNA silencing in human osteoblasts [19,46]. Additional evidence came from a study showing the importance of CYP27B1 for proliferation and osteogenic differentiation of human mesenchymal stromal cells (MSCs) [128,129]. MSCs of older donors had reduced *CYP27B1* expression and resistance to 25(OH)D_3_ regulation of osteoblast differentiation [130]. Broader tissue distribution of extra renal CYP27B1 expression beyond bone was recently summarized by Bikle et al. [131]. 

However, renal synthesis is still considered the major contributor to circulating 1α,25-OH_2_D_3_ levels. Only in diseases such as sarcoidosis extra is renal synthesis sufficient to contribute to circulating levels. The presence of 1α,25-OH_2_D_3_ synthesis within bone provides a means to explain the associations of bone phenotypes and other parameters with circulating 25(OH)D_3_ and not with 1α,25-OH_2_D_3_, as discussed by Anderson and colleagues [132,133]. Pharmacokinetic differences between locally produced 1α,25-OH_2_D_3_ from 25(OH)D_3_ and added 1α,25-OH_2_D_3_ have been suggested from a cell culture study [134]. Further studies, in particular, in vivo studies, are needed for full appreciation of the impact of an autocrine/paracrine role of 1α,25-OH_2_D_3_.

Observations that the vitamin-D-binding protein receptors cubulin and megalin, as well as the vitamin D_3_ 25-hydroxylase genes *CYP2R1* and *CYP3A4*, are also expressed in human osteoblasts, supports an autocrine/paracrine role [19,30,131].

Renal CYP27B1 is tightly controlled by factors such as parathyroid hormone (PTH) and fibroblast growth factor-23 (FGF23), which are involved in calcium and phosphate homeostasis. Extrarenal CYP27B1 expression is differently regulated, and probably involves other factors and tissue specificity [135]. For example, PTH and ambient calcium do not regulate *CYP27B1* in human osteoblasts [30], while 1α,25-OH_2_D_3_ reduces *CYP27B1* expression in human MSCs similar as in the kidney [136]. Several growth factors and cytokines can regulate CYP27B1 expression. IGF-I increases *CYP27B1* expression in human MSCs [136]. Interleukin-1 stimulates while interferon-β reduces *CYP27B1* expression in human osteoblasts [30,69]. The earlier described impact of the osteoblast differentiation stage on 1α,25-OH_2_D_3_ action can also be translated to expression of CYP27B1. *CYP27B1* expression is increased by 25(OH)D_3_ in human MSCs [136], but not in mature osteoblasts [30].

### 4.2. CYP24A1

The first step in the degradation cascade of 1α,25-OH_2_D_3_ is hydroxylation at the C-24 position by 24-hydroxylase (CYP24A1) [137]. CYP24A1 is expressed in all vitamin D target cells, and its expression is very rapidly and strongly increased after 1α,25-OH_2_D_3_ binding to VDRs [138,139,140,141]. The VDR level is tightly linked to the induction of CYP24A1 expression and 24-hydroxylase activity and, consequently, degradation of 1α,25-OH_2_D_3_. Thus, the homologous upregulation of VDRs concomitantly induces the inactivation of 1α,25-OH_2_D_3_, and thereby limits its effect [142,143]. Hydroxylation at the C-24 position of 1α,25-OH_2_D_3_ or 25(OH)D_3_ alone does not immediately lead to an inactive vitamin D molecule. Henry and Norman demonstrated in the 1970s the functional significance of 24,25(OH)_2_D3 for normal chicken egg hatchability and calcium and phosphorus homeostasis [144,145]. The effects of 24,25(OH)_2_D_3_ on bone metabolism were shown in human, chicken, rat, and mouse studies. 24,25(OH)_2_D_3_, synergistically with PTH, directly stimulates mineralization, and 24,25(OH)_2_D_3_ decreases the number and size of resorption sites on the bone surface [146,147]. 24,25(OH)_2_D_3_ restores and accelerates the bone mineral apposition rate in vitamin-D-deficient and in parathryoidectomized rats [147]. 24,25(OH)_2_D_3_ did not change bone histomorphometric parameters in ovariectomized rats [148], but 24,25(OH)_2_D_3_, and not 1α,25-OH_2_D_3_, increased bone strength [149].

Several studies focused on 24-hydroxylated vitamin D molecules and fracture healing. 24,25(OH)_2_D_3_ binds to fracture calluses [150], and improves fracture healing [151,152,153]. Serum 24,25(OH)_2_D_3_ levels were found to correlate with fracture healing in chicken [151], but not in a small human study in 1978 [154]. However, a study on pre-dialysis renal insufficiency patients supported a direct, i.e., PTH-independent, functional role of 24,25(OH)_2_D_3_ in human bone. 24,25(OH)_2_D_3_, together with 1α,25-(OH)_2_D_3_, preserved the osteoblast perimeter and improved mineralization, while 1α,25-(OH)_2_D_3_ alone was ineffective [155]. A direct effect on bone, in particular osteoblasts, is supported by in vitro studies showing that, similarly to 1α,25-OH_2_D_3_, 24,25(OH)_2_D_3_ has direct effects on human osteoblast differentiation [45]. Knowing that 24-hydroxylation per se does not lead to inactivation of vitamin D molecules, it is important to understand target tissue/target cell dynamics of the next steps in the degradation cascade. Control of the velocity of the subsequent steps in the degradation pathway can be a means to regulate vitamin D action in target tissues/cells. Together, these data on CYP24A1 and the biological activities of 24,25(OH)_2_D_3_ add to the notion of an auto/paracrine vitamin D regulatory system in bone. This system is most likely not restricted to bone and may also be present in other tissues. 

## 5. Conclusions

This review revealed that the central role for vitamin D in bone physiology is directed via osteoblasts and depends on their stage of development. VDRs and the vitamin-D-metabolizing enzymes CYP27B1 and CYP24A1, known from the vitamin D endocrine system, are present and functional in osteoblasts. This uncovers a direct local role for 1α,25-OH_2_D_3_ vitamin D in osteoblast function, and expands the vitamin D action profile from endocrine regulation of calcium and phosphate homeostasis to an auto/paracrine regulatory network in bone. Several target-tissue-derived factors (growth factors, cytokines), intracellular signaling cascades (Wnt), and functional states of the osteoblast interact with this auto/paracrine network and determine the eventual response. In this way, vitamin D controls the proliferation, apoptosis, differentiation, and mineralization of osteoblasts, as well as their gene profile and interaction with other factors that maintain healthy bone. Moreover, even local degradation products of vitamin D metabolism (24,25(OH)_2_D_3_) have a beneficial contribution to osteoblast function. Together, these observations underscore the importance of contextual knowledge (molecular and cellular) in order to fully understand and appreciate the effects of vitamin D on bone cells.

This warrants research for the next 100 years: future studies may focus on assessing tissue levels of vitamin D metabolites in addition to circulating levels, and study functionality of the complete metabolic profile of vitamin D.

## Figures and Tables

**Table 1:** Number of transcripts on average that are 2-fold up- or downregulated in the pre-mineralization or mineralization phase of human osteoblasts *.

Condition	# of Genes UP	# of Genes DOWN
Pre-mineralization phase	155	164
Mineralization phase	166	236
In both phases	10	8

* Experimental procedures and culture conditions of human osteoblasts (SV-HFO) are described in Woeckel et al. [99]. Two-fold change is based on the average expression at the timepoints in the pre-mineralization or mineralization period.

**Table 2:** Gene symbols of transcripts that are 2-fold upregulated or downregulated in both the pre-mineralization and mineralization phases of human osteoblasts (i.e., 10 and 8 in both phases in Table 1) *.

Upregulated	Downregulated
*ABCC3*	*AGAP10*
*CYP24A1*	*CCL20*
*MAGEE1*	*DDIT3*
*RARRES2*	*GRK4*
*RICH2*	*LOC727869*
*SLC25A45*	*NFE2L2*
*SULT1C2*	*ODF1*
*THBD*	*TSC22D2*
*TMEM180*	
*TOX3*	

* Experimental procedures and culture conditions of human osteoblasts (SV-HFO) are described in Woeckel et al. [99]. Two-fold change is based on the average expression at the timepoints in the pre-mineralization or mineralization period.

**Table 3:** Number of transcripts uniquely 2-fold up- or downregulated in either the pre-mineralization or in the mineralization phase of human osteoblasts *.

Condition	# of Genes UP	# of Genes DOWN
Pre-mineralization phase	65	66
Mineralization phase	77	100

* Experimental procedures and culture conditions of human osteoblasts (SV-HFO) are described in Woeckel et al. [99]. The 2-fold and 0.8–1.2-fold change is based on the average expression at the timepoints in the pre-mineralization or mineralization period.

**Table 4:** Overview of transcript gene names that are uniquely 2-fold up- or downregulated in either the pre-mineralization or mineralization phase of human osteoblasts *.

Pre-Mineralization Phase				Mineralization Phase			
Upregulated		Downregulated		Upregulated		Downregulated	
AQR	RAB9BP1	ADAM22	RARA	ABCD4	MYH11	AASDH	MOSPD1
ARHGEF7	RLTPR	ADORA1	RBM	AKAP13	NFIX	ABCD3	MRPS23
ATAT1	SARDH	ATF7IP2	RIMKLB	ANKRD11	ORC5L	ABT1	MS4A1
ATG16L1	SHISA8	BAGE	SLC19A1	APIP	PCDHB3	ACTR3C	MTUS2
ATP1A4	SLC38A11	BRS3	SLC26A7	ARHGDIB	PDLIM5	ANUBL1	NCRNA00188
BCL11A	SZT2	BRWD1	SLC3A1	ASH1L	PDZRN4	AP5S1	NDRG2
BMF	TEX9	BST2	SNRPN	ATM	PGAP1	B4GALNT2	NDUFB7
BMP15	TMEM120B	C1orf68	TBK1	BNC2	PLEKHG2	C11orf65	NRAP
C15orf48	TMEM33	CACNA1A	TFAP4	BPTF	PPP4R4	C14orf156	NUDT14
C2orf27A	UBE2G2	CCDC144C	THPO	BRD4	PRPF18	C14orf2	OGFR
C3orf20	UBXN10	CSF2RA	TMPRSS15	CAP1	PTGES	C17orf104	PANK2
C8orf34	UNC13C	CTNS	TRIB3	CCDC67	PTGS1	C4orf36	PAPPA
CCDC124	ZC3H12A-DT	DEFB132	TRMT2A	CCDC76	RAB3IP	CCL5	PAX8
COL24A1	ZNF668	EDA	TTBK2	CD14	RASAL2	CCT2	PIP5K1A
CTU2	ZNF703	ERCC6L2	ZNF396	CLCN4	RG9MTD2	CNOT2	PLCH1
DCTN2		FAM219A	ZNF93	CROCCL1	SERTAD4	COX7C	PMCH
DOCK6		FCGR2C		DCLK3	SMARCA4	CSRP2BP	PML
DST		FLJ10213		DPP4	SRGAP1	DAZL	POLE4
DUSP28		FSD1L		EGFR	SRRM2	DBI	POLR2K
EPG5		GAS2		EP300	SULF1	DCUN1D1	PTPRA
EYA2		GLIPR1		FAM102A	TBC1D13	DNAH1	RHEB
GABRB3		GPR155		FAM186A	TBL1X	DUSP16	RPAIN
GNRHR		HM13		FAM20C	UGGT2	EEF1D	RPL13
HCRTR2		ICA1		FGF7	VCAN	EGFL8	RPL14
HIST1H4C		KLHL36		FLJ11292	ZNF397	EHD1	RPL34
HSPB7		KLK7		FLJ13773	ZNF462	ELP6	RPS11
IL1RN		LEKR1		FOXP2	ZSWIM1	ESPNL	SEMA6D
KCNJ15		LELP1		GABRA5		EXOG	SHLD1
LOC100131283		LIN28B		H2AFY		EXOSC2	SLC10A7
LOC148987		LOC100286895		HMCN1		FABP4	SLC9A5
LOC149351		LOC100287114		HOXA6		FAM126A	SNAP23
LOC285205		LOC283854		HSPA12A		FAM27A	SNCAIP
LOC645591		LOC285692		IL17C		FAXC	SNTG1
LOC728903		LOC390595		INTS4		FUT7	STEEP1
LOC780529		LOC440944		KCNAB1		GOSR1	STK32A
LRRC46		MAN1A2		KCNG3		GPR39	STMN3
LYZL6		MAPRE3		KRTAP3-3		GSN	SUPT16H
MGC42157		MGC12916		LOC100127980		HCG4P6	TAL1
MRS2		MRPL19		LOC100128640		IRGQ	TBC1D8
NCOR2		MSR1		LOC100131993		KCNIP3	TEN1
NOX4		MYO10		LOC283682		KY	TLK1
NTRK2		NR2E3		LOC285500		LOC100133109	TWF1
OR1J4		NUP210L		LOC388210		LOC100287911	TXNIP
PDE1A		OTX2		LOC441461		LOC100289246	UHRF1BP1L
PENK		PCLO		MAGEB18		LOC338862	UQCRB
PGM2L1		PKP2		MARK2		LOC643749	UQCRQ
PHC3		PLXNA2		MEGF10		LPAR5	VMA21
POU2F1		POU2F2		MGAT5B		MATR3	WFDC21P
PRRG2		PRLR		MLXIP		MMP16	XAF1
PTCD3		RAD54L2		MS4A6A		MMP17	ZNF880

* Experimental procedures and culture conditions of human osteoblasts (SV-HFO) are described in Woeckel et al. [99].

## Data Availability

Not applicable.

## Supplementary Materials

The following supporting information can be downloaded at https://www.mdpi.com/article/10.3390/nu15030480/s1, Figure S1: Literature search strategy performed October 2022.
