# Supplementary material for: Vitamin D and Bone: A Story of Endocrine and Auto/Paracrine Action in Osteoblasts
Source: Nutrients. 2023 Jan 17;15(3):480. doi: 10.3390/nu15030480 (PMC9919888; doi:10.3390/nu15030480)
Supplement: Supplementary file 1 [file nutrients-15-00480-s001.zip › nutrients-2105133-supplementary.pdf]

Figure S1: Literature search strategy performed October 2022

Data base searched	Years of coverage	Records	Records after duplicates removed
Medline ALL Ovid	2016 - present	2067	2060
Embase.com	2016 - present	2185	653
**Total**		**4252**	**2713**
Excluded			2583
Included			**128**
Retracted			2

Medline ALL Ovid 2067

(* exp Vitamin D / OR * Calcitriol / OR * Receptors, Calcitriol / OR * Vitamin D Deficiency / OR * Vitamin D-Binding Protein / OR (((vitamin* OR hydroxyvitamin* OR dihydroxyvitamin*) ADJ (D OR D3)) OR colecalciferol* OR cholecalciferol* OR ergocalciferol* OR Calcitriol*).ti.) AND (* exp "Bone and Bones"/ OR Osteoblasts/ OR Osteocytes/ OR Osteoclasts/ OR Fibroblast Growth Factors / OR Extracellular Matrix / OR exp Bone Diseases/ OR Hypercalcemia / OR Receptors, Fibroblast Growth Factor / OR Calcification, Physiologic / OR (bone OR bones OR skeleton* OR skeletal* OR fibroblast-growth-factor* OR extracellular-matri* OR extra-cellular-matri* OR Mineralizat* OR Mineralisat* OR demineralizat* OR demineralisat* OR Osteoblast* OR Osteoclast* OR Osteocyt* OR Fracture* OR FGF23 OR hypercalcemi* OR hypercalcaemi* OR (disc ADJ3 degenerat*) OR craniosteno* OR scolios* OR Osteoarthrit* OR spondyloarthrit* OR spondylarthrit* OR coxarthrit* OR gonarthrit* OR calcificat* OR Osteoporos* OR ricket* OR (ankylosing ADJ3 spondylitis) OR arthritis OR acromegal*).ti.) AND (2016 OR 2017 OR 2018 OR 2019 OR 2020 OR 2021 OR 2022 OR 2023).yr. NOT (letter* OR news OR comment* OR editorial* OR congres* OR abstract* OR book* OR chapter* OR dissertation abstract*).pt. AND english.la. NOT (exp animals/ NOT humans/)

Embase.com 2185

('vitamin D'/exp/mj OR 'vitamin D receptor'/exp/mj OR 'vitamin D deficiency'/exp/mj OR 'vitamin D binding protein'/de/mj OR 'vitamin d supplementation'/de/mj OR (((vitamin* OR hydroxyvitamin* OR dihydroxyvitamin*) NEXT/1 (D OR D3)) OR colecalciferol* OR cholecalciferol* OR ergocalciferol* OR Calcitriol):ti) AND (bone/mj/exp OR 'bone cell'/mj/exp OR 'bone tissue'/mj/exp OR 'bone injury'/mj/exp OR 'fibroblast growth factor'/mj/exp OR 'extracellular matrix'/mj/exp OR 'bone metabolism'/mj/exp OR 'calcium bone level'/mj/de OR 'bone microarchitecture'/mj/exp OR 'bone malformation'/mj/exp OR 'bone disease'/mj/exp OR hypercalcemia/mj OR 'fibroblast growth factor receptor'/mj OR 'bone disease'/exp/mj OR calcification/mj OR (bone OR bones OR skeleton* OR skeletal* OR fibroblast-growth-factor* OR extracellular-matri* OR extra-cellular-matri* OR Mineralizat* OR Mineralisat* OR demineralizat* OR demineralisat* OR Osteoblast* OR Osteoclast* OR Osteocyt* OR Fracture* OR FGF23 OR hypercalcemi* OR hypercalcaemi* OR (disc NEAR/3 degenerat*) OR craniosteno* OR scolios* OR Osteoarthrit* OR spondyloarthrit* OR spondylarthrit* OR coxarthrit* OR gonarthrit* OR calcificat* OR Osteoporos* OR ricket* OR (ankylosing NEAr/3 spondylitis) OR arthritis OR acromegal*):ti) AND [2016-2023]/py NOT ([Conference Abstract]/lim OR [Letter]/lim OR [Note]/lim OR [Editorial]/lim) AND [english]/lim NOT ([animals]/lim NOT [humans]/lim)
